# Optimizing microbiological surveillance during selective digestive decontamination in the intensive care unit: an *in silico *simulation study

**DOI:** 10.1186/s13054-025-05494-5

**Published:** 2025-06-17

**Authors:** Jelle L. G. Haitsma Mulier, Fleur J. van Dijk, Valentijn A. Schweitzer, Marc J. M. Bonten, Lennie P. G. Derde, Olaf L. Cremer

**Affiliations:** 1 Julius Centre for Health Sciences and Primary Care, University Medical Centre Utrecht, Utrecht, The Netherlands; 2 Department of Intensive Care Medicine, University Medical Centre Utrecht, Utrecht, The Netherlands; 3 Department of Medical Microbiology, University Medical Centre Utrecht, Utrecht, the Netherlands; 4 European Clinical Research Alliance on Infectious Diseases (Ecraid), Utrecht, The Netherlands

**Keywords:** Selective digestive decontamination, Intensive care unit, Microbiological surveillance, Cultures, Costs, Simulation

## Abstract

**Background:**

Selective Digestive Decontamination (SDD) prevents infections and reduces mortality in the intensive care unit (ICU). Microbiological surveillance is considered essential for effective decontamination and detecting antibiotic resistance. However, its optimal frequency is unclear. We compared microbiological yield and costs of different surveillance intervals during SDD.

**Methods:**

In a computational simulation study, using data from a Dutch ICU, three surveillance scenarios were compared: (A) twice-weekly, (B) once-weekly, and (C) no surveillance. The primary outcome was the number of clinically relevant potentially pathogenic microorganisms (PPMs) detected per scenario. Secondary outcomes included detection of colonisation persistence prompting SDD intensification and surveillance costs.

**Results:**

We included 8,499 ICU admissions, 52,553 clinical and 75,567 SDD cultures. Scenario A yielded 911 (95% CI 905–917) PPMs per 1,000 days, of which 90 (88–94) were clinically relevant: 9 (9–10) multidrug-resistant microorganisms, 68 (66–71) microorganisms resistant to standard therapy, and 13 (12–14) infection-related microorganisms. Scenarios B and C yielded 85 (82–88) and 77 (75–80) relevant PPMs, respectively (94% and 86% compared to scenario A). Scenario A identified 56 (55–58) cases of colonisation persistence per 1,000 days while scenarios B and C detected 43 (42–45) and 12 (11–12), respectively. Total costs of SDD surveillance were €78,774, €55,208, and €31,522 per 1,000 days for scenarios A, B and C.

**Conclusion:**

Compared to twice-weekly surveillance, once-weekly microbiological surveillance reduces costs by 30% with 6% loss in clinically relevant PPM detections. No surveillance reduces costs by 60% with 14% detection loss.

**Supplementary Information:**

The online version contains supplementary material available at 10.1186/s13054-025-05494-5.

## Introduction

ICU-acquired infections are common and contribute to increased morbidity, mortality and healthcare costs [1, 2]. Most of these infections are of endogenous origin, caused by potentially pathogenic microorganisms (PPMs) that patients carry in their throat or gut [3, 4]. These PPMs are usually aerobic, whereas infections are rarely caused by the normally predominant anaerobic flora [2, 5–7].

Over the past decades, many approaches have been adopted to prevent ICU-acquired infections. One of these is the use of Selective Digestive Decontamination (SDD), a prophylactic regimen with antibacterial and antimycotic agents that selectively targets PPMs colonising the digestive tract, while preserving the anaerobic flora [8–10]. SDD encompasses four-times daily topical administration of an oral paste and enteral suspension containing colistin, tobramycin, and nystatin or amphotericin. This is combined with four days of systemic prophylaxis with a 3rd generation cephalosporin (usually ceftriaxone or cefotaxime) to empirically target primary endogenous infections that may already be incubating at the time of ICU admission [2, 7, 10].

Application of SDD in ICU patients was first studied in 1984. In that study, the overall infection rate among trauma patients decreased from 81 to 16% [2]. A number of large, cluster-randomized trials and meta-analyses of individual-based randomized trials have since provided robust evidence that SDD improves patient outcomes [1, 11, 12]. Consequently, national guidelines in the Netherlands now recommend SDD for ICU patients who are expected to receive mechanical ventilation for > 48 h or have an admission duration of > 72 h [1].

Since its first conception, microbiological surveillance has been marked as a key component of SDD [4, 13, 14]. It is considered essential for detecting the acquisition of resistant microorganisms and for monitoring the effectiveness of decontamination itself, with subsequent protocolized adjustments in the SDD regimen when colonisation persistence (or recurrence) is detected [2, 3, 7, 9, 15]. However, the effectiveness of these interventions remains uncertain [16, 17].

In the first SDD study in the ICU, microbiological surveillance was performed three times per week and involved culturing of the rectum, urine, throat, sputum, and wounds [2]. Subsequent studies have varied greatly in their approach to microbiological monitoring, ranging from no surveillance at all [18–20], to daily surveillance [21]. Although there is no evidence on the optimal frequency of microbiological surveillance, over 90% of Dutch hospitals adhere to a twice-weekly microbiological surveillance protocol [1, 16]. In a cost-effectiveness study, the costs for microbiological cultures represented a substantial portion of SDD expenditures [22]. Since SDD is used in a growing number of ICUs worldwide, culturing practices could have considerable economic impact [23]. Therefore, we quantified the microbiological yield and costs of different surveillance strategies in a computational *(in silico)* simulation study based on retrospectively collected clinical data.

## Methods

### Study population

This study was performed in the 32-bed mixed medical-surgical ICU of the University Medical Centre Utrecht (UMCU), a tertiary care hospital in The Netherlands. Adults admitted between July 2011 and December 2022 who received at least one administration of topical antimicrobial paste or suspension, had SDD culture data available, and underwent SDD admission cultures were eligible for analysis. Each ICU admission was considered separately, allowing multiple inclusions per patient.

### SDD protocol

SDD was recommended for patients expected to require > 48 h of mechanical ventilation or > 72 h of ICU stay. SDD cultures of rectum, throat and sputum were obtained on admission, followed by twice-weekly surveillance on Mondays and Thursdays. Throat and sputum cultures were incubated on blood, chocolate, and yeast agar plates for two days at 35–37 ˚C and screened for respiratory PPMs, Gram-negative bacteria, and yeasts. Rectum cultures were incubated on blood, MacConkey, and yeast agar plates for two days at 35–37 ˚C and screened for Gram-negative bacteria, haemolytic streptococci, *S. aureus*, and yeasts. Species identification was performed using the Bruker Microflex MALDI mass spectrometer. Susceptibility testing was performed using BD Phoenix™.

The enteral suspension and oral paste used for SDD were administered every 6 h and contained colistin and tobramycin (directed against Gram-negative PPMs) and amphotericin B or nystatin (targeting fungi and yeasts). A four-day course of systemic prophylaxis was provided with either cefotaxime (1 gram every 6 h) or ceftriaxone (2 g once daily). If surveillance cultures indicated persistent or recurrent colonisation with PPMs, the protocol advised intensification of the topical SDD application to eight times daily, and/or the initiation of nebulisations with tobramycin, colistin, or amphotericin B, based on culture results (Figure S1). The SDD protocol remained unchanged throughout the study period.

### Study design

We performed an in silico simulation study to compare three SDD surveillance strategies. Scenario A reflected current practice, with admission cultures followed by twice-weekly surveillance of the throat, rectum, and sputum. Scenario B included admission cultures and once-weekly surveillance. Scenario C included only admission cultures without subsequent microbiological surveillance.

The primary outcome was the number of clinically relevant PPMs identified per scenario (analysing both SDD and clinical cultures). Clinical relevance was assessed, distinguishing between three levels: (1) direct relevance (including MRSA, VRE, ESBL-producing Enterobacteriaceae or other multidrug-resistant organisms (MDRO) necessitating immediate barrier precautions, (2) high relevance (encompassing microorganisms resistant to third-generation cephalosporins or fluconazole, including both intrinsically resistant microorganisms and microorganisms with acquired resistance), and (3) infection-related PPMs (referring to microorganisms adjudicated as the causative pathogen of an ICU-acquired infection at the time of sampling or later during ICU admission).

Only new findings were included in this outcome. New findings were defined as microorganisms not previously cultured during the same ICU stay. Identical species with differing susceptibility profiles were considered new findings. If surveillance and clinical cultures collected on the same day yielded the same isolate, the SDD culture was considered non-informative. Likewise, repeated detections of the same isolate at multiple surveillance sites were included as a new finding only once.

As a secondary outcome, we analysed detection of colonisation persistence that would necessitate intensified SDD administration. This was defined as the detection of a PPM after ≥ 4 days of SDD application that, according to protocol, would trigger intensification of SDD administration (Figure S1).

Lastly, we determined the SDD culture costs of each scenario. Other cultures performed on clinical indication were not included in this analysis. The cost per culture was calculated as the sum of order fees, culture plate expenses, and laboratory costs for species identifications and resistance tests. To this end, microbiological proced ure registration codes for each patient were multiplied by the corresponding hospital culture tariffs, adjusted to 2023 price levels [24].

### Data collection

Data were extracted from electronic health records (HiX, ChipSoft; MetaVision, iMDsoft) and the laboratory information system GLIMS (Clinisys). Infection episodes, including adjudication of causative pathogens, had been prospectively recorded as part of the Molecular Diagnosis and Risk Stratification of Sepsis (MARS) project [25].

### Data analysis

In scenario A, all available microbiological data were analysed. Scenario B simulated once-weekly surveillance by randomly removing SDD culture data from Mondays or Thursdays per patient. Scenario C considered only admission cultures, thus excluding all SDD surveillance data. In all three scenarios, clinical cultures were retained. To allow extrapolation to other centres, we report the rates of relevant findings per 1,000 ICU days. We performed bootstrapping (1,000 iterations) to calculate the 95% confidence interval (CI) for the number of relevant PPMs per scenario. The same procedure was performed to obtain the number of PPMs triggering SDD intensification in each scenario. Additionally, we quantified the number of missed and delayed detections in scenarios B and C compared to scenario A.

Only patients with complete cost data for SDD cultures were included in the cost analysis. Cost data were unavailable for patients admitted before 2013. Only 0.2% of SDD cultures from patients admitted in or after 2013 had missing cost data; these were imputed using the median cost of a positive or negative SDD culture. Bootstrapping was performed to obtain 95% CIs.

We performed subgroup analyses for cost-effectiveness by admission type, immunocompromised status, COVID-19 status, gastro-intestinal surgery, positive versus negative admission cultures, and positive versus negative first surveillance cultures. To test the robustness of our findings, we performed a sensitivity analysis focused exclusively on ICU admissions during the COVID-19 pandemic (regardless of COVID-19 status). All analyses were performed using R version 4.3.1 [26].

## Results

**Fig. 1:**
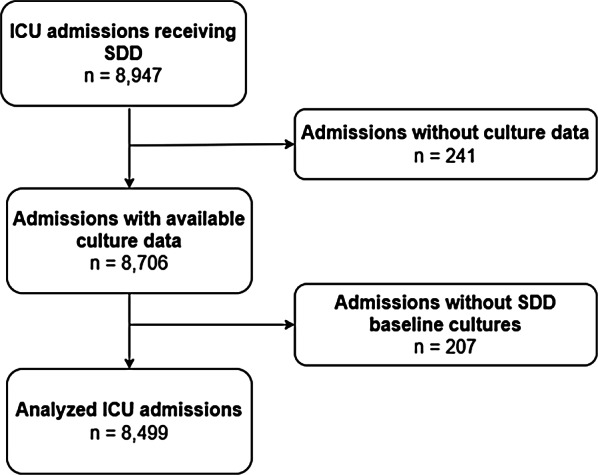
Flowchart of inclusion

From July 2011 to December 2022, 8,947 ICU admissions received SDD, of whom 8,499 were included in the analysis (Fig. 1). Virtually all patients (96%) received invasive mechanical ventilation, and a majority (76%) also required vasopressors during their stay in ICU (Table 1).

**Table 1:** Characteristics of the included cohort

	Presented as	*n* = 8,499
Age	Median (IQR)	61 (50–70)
Sex at birth		
Female	n (%)	3,133 (37)
Admission type		
Medical	n (%)	4,757 (56)
Surgical elective	n (%)	1,272 (15)
Surgical emergency	n (%)	2,470 (29)
ICU readmission	n (%)	984 (12)
Severity of disease scores		
APACHE IV	Median (IQR)	78 (60–101)
SOFA score at admission	Median (IQR)	7 (5–9)
Comorbidities		
Hypertension	n (%)	2,486 (29)
Any malignancy	n (%)	1,455 (17)
Immune deficiency	n (%)	1,344 (16)
Diabetes	n (%)	1,279 (15)
COPD or chronic respiratory insufficiency	n (%)	1,286 (15)
Congestive heart failure	n (%)	1,042 (12)
Myocardial infarction	n (%)	859 (10)
Chronic renal insufficiency	n (%)	579 (7)
Oxygen or ventilator support at home	n (%)	347 (4)
Organ support during admission		
Mechanical ventilation at any time	n (%)	8,177 (96)
Vasopressor use at any time^1^	n (%)	6,462 (76)
ICU admission duration in days	Median (IQR)	5.0 (2.3–10.6)
ICU mortality	n (%)	1,602 (19)

^1^As indicated by a cardiovascular SOFA score ≥ 3 at any time during admission

**Fig. 2:**
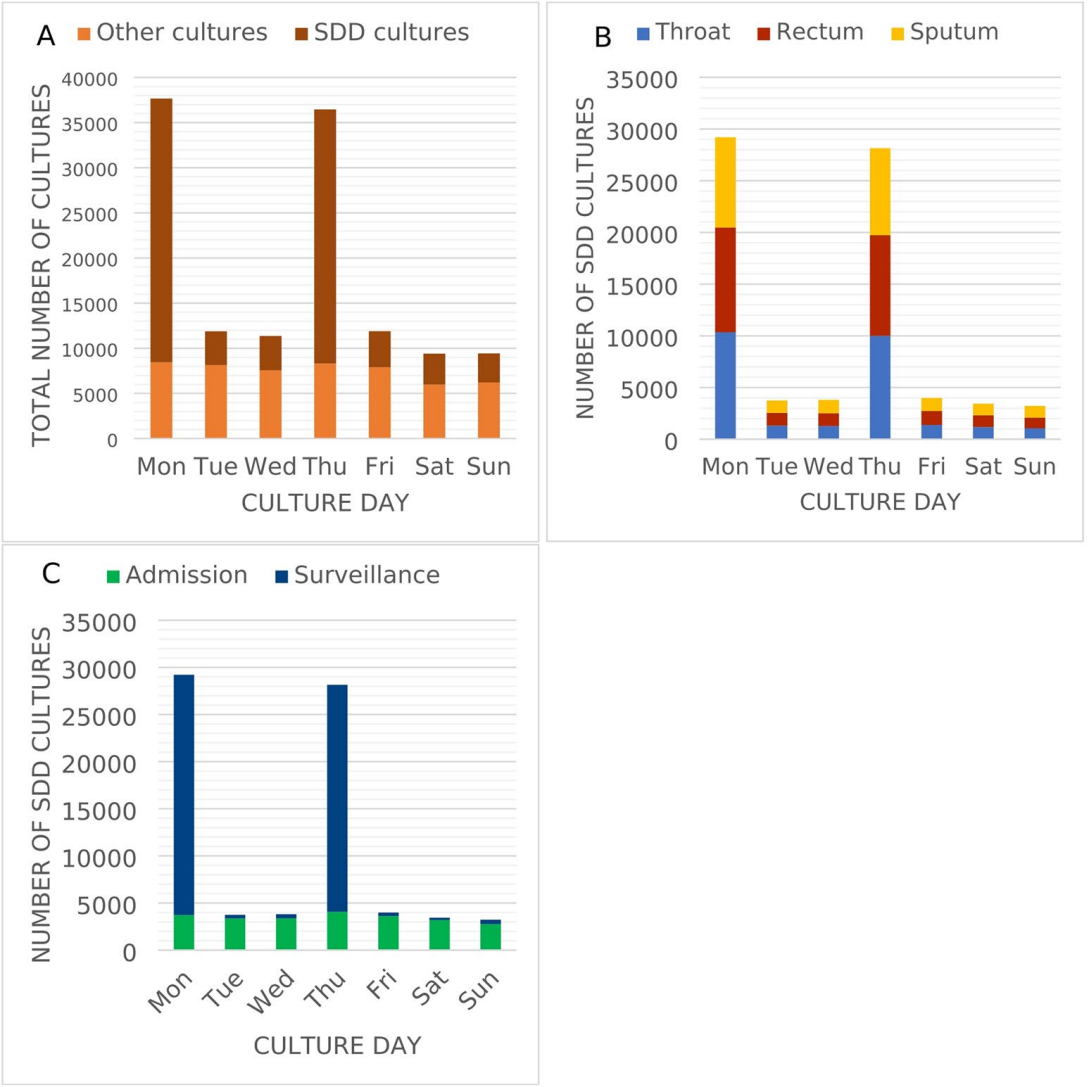
Distribution of microbiological cultures collected by day of the week. **A**: Distribution of SDD cultures vs. cultures performed for another indication (e.g., clinical cultures, MDRO screening); **B**: Distribution of SDD admission. cultures vs. SDD surveillance cultures (excluding clinical cultures); **C**: Distribution of SDD cultures by culturing site (excluding clinical cultures).

A total of 128,120 cultures were obtained during 76,964 ICU days. Of these, 75,567 (59%) were collected as part of SDD (24,066 (32%) admission cultures and 51,501 (68%) surveillance cultures) and remaining samples were obtained at the discretion of attending physicians (Fig. 2A and C). Sputum, throat, and rectum cultures were evenly distributed among SDD cultures (Fig. 2B). Overall, 48,140 (38%) cultures were positive, yielding 70,103 PPMs. Of these, 49,935 (71%) PPMs were detected in SDD samples. Admission cultures had a higher positivity rate (62%) than surveillance (38%) or clinical cultures (26%).

### Primary outcome

**Fig. 3:**
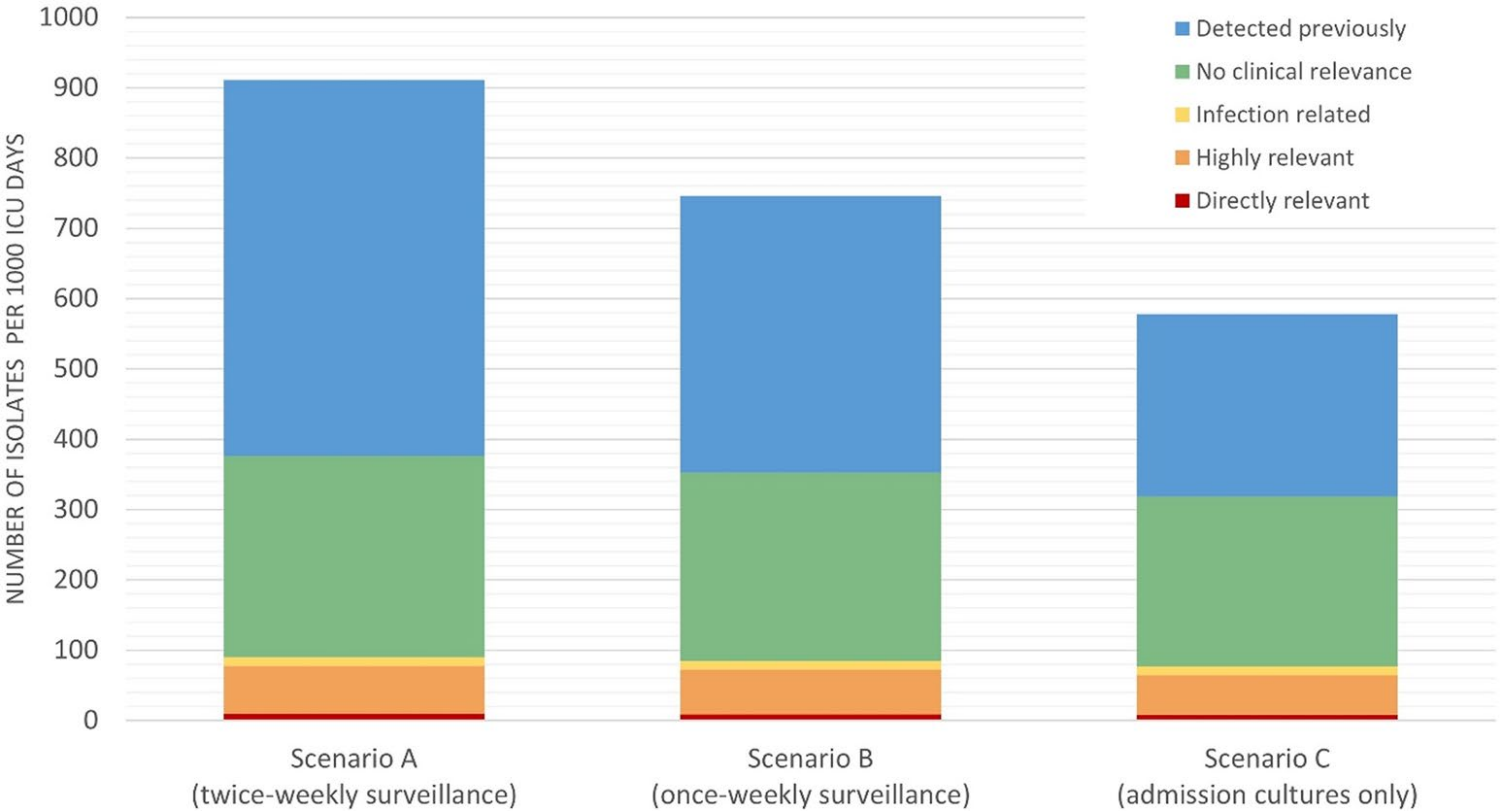
Detection rates per 1,000 ICU days and clinical relevance of potentially pathogenic microorganisms across different SDD surveillance scenarios

Scenario A yielded 911 (95% CI 905–917) PPMs per 1,000 ICU days. In scenarios B and C, detection rates were 746 (95% CI 741–751) and 591 (95% CI 573–582) PPMs per 1,000 ICU days, respectively. In scenario A, 90 (95% CI 88–94) PPMs per 1,000 ICU days (9.9%) were deemed clinically relevant, including 9 (95% CI 9–10) multidrug-resistant PPMs (direct relevance), 68 (95% CI 66–71) PPMs resistant to standard therapy (high relevance), and 13 (95% CI 12–14) infection-related microorganisms. In scenarios B and C, 85 (95% CI 82–88) and 77 (95% CI 75–80) clinically relevant PPMs per 1,000 ICU days were detected, corresponding to 94% and 86% of clinically relevant PPMs identified in scenario A (Fig. 3). A detailed overview of missed PPMs in scenario B and C, relative to scenario A, is provided in Table S1. The results of the sensitivity analysis in the COVID-19 period are available in the Supplementary materials.

### Delayed detections

Most of the clinically relevant PPMs identified in scenario A were detected on the same day in scenarios B and C. However, in scenario B, 2.9 (95% CI 2.6–3.4) clinically relevant PPMs per 1,000 ICU days were detected with a median delay of 3 (IQR 3–4) days, compared to 2.7 (95% CI 2.4–3.1) PPMs with a median delay of 4 (IQR 2–8) days in scenario C.

### Secondary outcomes

In scenario A, there were 56 (95% CI 55–58) cases of colonisation persistence per 1,000 ICU days that required SDD intensification. Most (92%) were detected through SDD surveillance samples. In scenarios B and C, we observed 43 (95% CI 42–45) and 12 (95% CI 11–12) such triggers per 1,000 ICU days, respectively. Of the triggers identified in scenario A, 55.7% were detected on the same day in scenario B, while an additional 21.3% were identified with a median delay of 3 (IQR 2–5) days. In scenario C we observed 7.8% of triggers on the same day as in scenario A and an additional 12.6% with a median delay of 5 (IQR 3–7) days. Of note, clinical adherence to intensification triggers varied substantially in our cohort. When intensification was indicated, nebulised antibiotics were prescribed in 73% of cases, while orabase and oral suspension regimens were intensified in only 35% and 11% of cases, respectively.

Cost data were available for 7,231 ICU admissions, representing 85.1% of the total cohort (Table 2). The median expenditure for microbiological cultures was €734 (IQR €407–1387) per ICU admission (€480 (IQR €287–825) for SDD cultures and €280 (IQR €97–711) for clinical cultures). The median costs per SDD culture were higher for admission samples than for surveillance samples (€68 (IQR €54–134) versus €54 (IQR €54–68), respectively). SDD culture costs were attributable to order fees and culture plates (60%), expenses for species determination (14%) and susceptibility testing (26%). The total expenditure for SDD surveillance was €78,774 (95% CI €76,810 − 80,884) per 1,000 days in scenario A, compared to €55,208 (95% CI €54,145 − 56,374) and €31,522 (95% CI €31,124 − 31,933) in scenarios B and C, respectively; representing overall cost reductions of 30% and 60%. The cost per clinically relevant isolate — analogous to a “number needed to treat”— was €1,453 (95% CI €1407–1503) in scenario A, €1,199 (95% CI €1158–1242) in scenario B, and €824 (95% CI €793–859) in scenario C. Subgroup analyses yielded similar findings (Table 3; Table S2).

**Table 2:** Costs associated with different SDD surveillance scenarios (SDD cultures only)

	Scenario A Admission + twice weekly surveillance	Scenario B Admission + once weekly surveillance	Scenario C Admission, no surveillance
Number of ICU admissions	7,231	7,231	7,231
Number of cultures	64,229	42,450	20,511
Total costs (95% CI)	€5,034,292 (€4,908,789 **–** €5,169,139)	€3,528,246 (€3,460,304 – €3,602,721)	€2,014,481 (€1,989,086 – €2,040,795)
Median costs			
Median costs per ICU admission (IQR)	€480 (€287 – €825)	€386 (€242 – €600)	€248 (€163 – €344)
Median costs per culture (IQR)	€54 (€54 – €79)	€54 (€54 – €120)	€68 (€54 – €134)
Mean costs			
Mean costs per ICU admission (SD)	€696 (€792)	€488 (€428)	€279 (€150)
Mean costs per culture (SD)	€78 (€53)	€83 (€57)	€98 (€66)

Costs represent the Dutch Healthcare Authority (NZa) maximum costs, indexed to 2023 tariffs.

**Table 3:** Costs per clinically relevant isolate for different subgroups

Subgroups^1^	ICU admissions *n* (%)^2^	Relevant findings^3^ *n* (%)^2^	Scenario A	Scenario B	Scenario C
			Costs per clinically relevant finding^4^	Costs per clinically relevant finding^4^	Costs per clinically relevant finding^4^
Complete cohort	7,231 (100)	3465 (100)	€1,453 (€1,407–1,503)	€1,199 (€1,158–1,241)	€824 (€793–859)
Medical admissions	4,054 (56)	1994 (57)	€1,466 (€1,400–1,533)	€1,207 (€1,151–1,264)	€814 (€775–857)
Acute surgical admissions	2,074 (29)	931 (27)	€1,450 (€1,367–1,546)	€1,217 (€1,144–1,314)	€899 (€832–972)
Elective surgical admissions	1,103 (15)	540 (16)	€1,406 (€1,299–1,541)	€1,137 (€1,038 − 1,258)	€743 (€677–823)
Immunocompromised patients	1,199 (17)	582 (17)	€1,473 (€1,359–1,596)	€1,162 (€1,066 − 1,267)	€766 (€704–844)
Patients with COVID-19	463 (6)	222 (6)	€1,866 (€1,641–2,138)	€1,454 (€1,266–1,703)	€776 (€677–916)
Gastrointestinal surgery	344 (5)	219 (6)	€1,133 (€995–1318)	€911 (€799–1,045)	€630 (€551–749)
Admission cultures positive	6,229 (86)	3,362 (97)	€1,355 (€1,309–1,403)	€1,111 (€1,069 − 1,153)	€768 (€739–798)
Admission cultures negative	1,002 (14)	103 (3)	€4,639 (€3,841–5,738)	€6,613 (€5,082 − 9,088)	-
First surveillance culture set positive^5^	2,191 (30)	1783 (51)	€1,563 (€1,490–1,649)	€1,227 (€1,165–1,302)	€636 (€601–678)
First surveillance culture set negative^5^	773 (11)	215 (6)	€3,431 (€3,047 − 3,971)	€2,853 (€2,478–3,358)	€1,496 (€1,280–1,803)

^1^With available culture costs data

^2^Relative to the complete cohort with available culture costs data

^3^Relevant findings in SDD cultures only

^4^Average costs for all SDD cultures (+ 95% CI), indexed to 2023 tariffs divided by the number of relevant isolates detected in these SDD cultures

^5^In the subgroup of patients with at least two surveillance culture sets (culture set = throat, sputum, rectum)

## Discussion

In this single-centre study involving 8,499 ICU admissions receiving SDD and 128,120 microbiological cultures, we found that once-weekly microbiological surveillance reduced costs by 30%, with a 6% decrease in clinically relevant PPM detections compared to twice-weekly surveillance. A strategy involving no surveillance reduced costs by 60% at a 14% detection loss.

Upon ICU admission, carriage with PPMs can be detected in most patients, while few acquire new carriage during ICU stay. In a recent study, 72% of ICU patients receiving SDD had Gram-negative bacteria in SDD cultures at ICU admission, with 24% of these patients maintaining carriage for 14 days post-admission [27]. In the current study, 87% of patients had positive admission cultures, and 38% of all surveillance cultures obtained during ICU-stay were positive. These findings suggest that surveillance cultures predominantly detect persistent carriage rather than new acquisitions, and thus that a reduction in culture frequency may be a reasonable approach.

Two prior studies have used once-weekly microbiological SDD surveillance in the ICU. The cluster-randomized SuDDICU trial recommended at least once-weekly microbiological surveillance but did not study its effects [19, 28]. Another study evaluating the endemicity of Pseudomonas aeruginosa in the ICU concluded that a once-weekly surveillance frequency was sufficient to detect nearly all patients at risk of *Pseudomonas aeruginosa* infection [29].

Reducing the frequency of surveillance cultures requires careful consideration of operational, clinical, and policy implications. Our study did not identify specific patient groups that would be at increased risk if the surveillance frequency were to be reduced. The decision on which surveillance strategy to follow must thus be made at the departmental level. The assessment of what is considered ‘acceptable’ and ‘safe’ will depend on the specific context of that department, considering factors such as the prevalence of MDRO’s and incidence of ICU-acquired infections.

A key concern is the potential impact of missed or delayed identification of clinically relevant isolates, which could lead to delayed diagnoses and poorer outcomes. However, in the current study, only 0.8% of all isolates detected in SDD cultures in scenario A were adjudicated to be infection-related, with 98% and 96% of those infection-related isolates also being detected in scenarios B and C, respectively, suggesting minimal impact on individual patient outcomes. Scenarios B and C resulted in 23% and 80% missed SDD intensification triggers, respectively. We consider a 23% loss acceptable, as the clinical benefits of interventions targeting persistent colonisation are likely limited. For instance, nebulized amphotericin B application for persistent yeast carriage only marginally accelerated eradication of Candida colonisation in mechanically ventilated patients without evidence of improved clinical outcomes [17]. Even if all triggers were followed up without delay and subsequent SDD adjustments would be fully effective, we expect that the overall mortality benefit of SDD would only be minimally be affected by a 23% reduction in SDD intensification triggers. Nonetheless, the clinical consequences of missed triggers cannot be inferred from our study. Given the substantial variability in how ICUs respond to indications for SDD intensification,^16^ further prospective research is warranted to evaluate the efficacy of various SDD intensification strategies and their impact on patient-centered outcomes. However, an 80% reduction in triggers, as in scenario C, seems too much for effective monitoring of colonisation persistence.

A previous cost-effectiveness analysis in Dutch ICUs concluded that SDD is a cost-saving strategy [22]. Our findings suggest that its cost-effectiveness could be further improved by reducing the frequency of microbiological surveillance. It is important to emphasize that potential costs associated with less frequent culturing, such as increased incidence of ICU-acquired infections, prolonged ICU stay, or increased reliance on other diagnostic tests, were not included in our estimates. However, we expect any additional expenses to be limited, given the low number of infection-related isolates observed. Additionally, our cost-saving estimates have been conservative, as only direct laboratory costs were considered, excluding other potential savings, such as reduced workload for nurses, less plastic waste, and lower carbon emissions.

Reducing the frequency of SDD cultures may lower the threshold for obtaining clinical cultures, thereby increasing diagnostic costs. It may also influence prescribing behaviour, such as encouraging the empirical use of antibiotics with broader antimicrobial coverage. Due to the retrospective nature of our study, these effects could not be directly quantified. However, the frequency of clinical cultures appears unaffected by the timing of SDD cultures, as clinical cultures were obtained with similar frequency on Monday and Thursday as on other days (Fig. 2A).

Strengths of our study include its large sample size and diverse study population, excluding only a small number due to missing data. This resulted in a representative sample of the mixed ICU population in countries with low MDRO prevalence. Although MDRO incidence rates are lower in the Netherlands than in many other countries, SDD is an intervention that is also mostly used in settings with low antimicrobial resistance. We provided transparent and reproduceable outcome definitions and applied a costing method based on publicly available prices, enabling other centres to compare their expenses to the costs reported in this study. Furthermore, complete and prospectively adjudicated clinical and infection registration data were available for all study patients, increasing the validity of our analysis. Lastly, we performed extensive subgroup analyses to evaluate the generalizability of our findings.

A key limitation of this study is its retrospective design. The reliance on simulations precludes precise predictions regarding the impact of missed relevant findings on patient outcomes such as ICU length of stay and mortality, and potential shifts in hospital expenditures. Additionally, culture results that were excluded in scenarios B and C may in reality have influenced clinical decision-making in the actual practice of scenario A. For instance, initiating antimicrobial treatment based on these cultures could have eradicated relevant isolates in subsequent cultures, potentially overestimating missed findings in scenarios B and C. Moreover, we deliberately adopted a liberal approach in adjudicating missed relevant cultures. For instance, pre-ICU cultures were not considered when determining whether an isolate was a new or relevant finding, potentially leading to an overestimation of new and relevant PPMs. Nonetheless, given the low proportion of PPMs with clinical relevance, and the overall small number of missed detections in scenarios B and C, we believe this limitation poses minimal risk to the validity of our findings. Finally, as our study was conducted in a setting with low antimicrobial resistance, its findings may not be generalizable to healthcare environments with a higher prevalence of multidrug-resistant organisms. In such settings, the diagnostic yield—and potentially the cost-effectiveness—of more frequent surveillance may be greater. To better assess the clinical implications of reduced surveillance frequencies and support implementation in diverse contexts, prospective studies—preferably multicenter and longitudinal—are warranted.

## Conclusion

In the ICU, once-weekly microbiological surveillance during SDD detects 94% of clinically relevant isolates, including antibiotic-resistant strains, and captures 77% of potential SDD intensification triggers, while reducing SDD-associated culturing costs by 30% compared to twice-weekly surveillance. Based on these findings, we consider once-weekly surveillance a viable and safe alternative in our setting. Limiting surveillance to admission cultures alone provides substantial additional cost savings (−60%) but results in a 14% loss in clinically relevant isolates and an 80% reduction in detected SDD intensification triggers.

## Electronic supplementary materials

Supplementary Material 1

## Data Availability

Study data and code are available upon reasonable request sent to the corresponding author.
