# Supplementary material for: Optimizing microbiological surveillance during selective digestive decontamination in the intensive care unit: an in silico simulation study
Source: Crit Care. 2025 Jun 17;29:246. doi: 10.1186/s13054-025-05494-5 (PMC12175364; doi:10.1186/s13054-025-05494-5)
Supplement: Supplementary file 1 — Supplementary Material 1 [file 13054_2025_5494_MOESM1_ESM.docx]

**Figure S1. Protocolized triggers for the intensification of SDD administration**


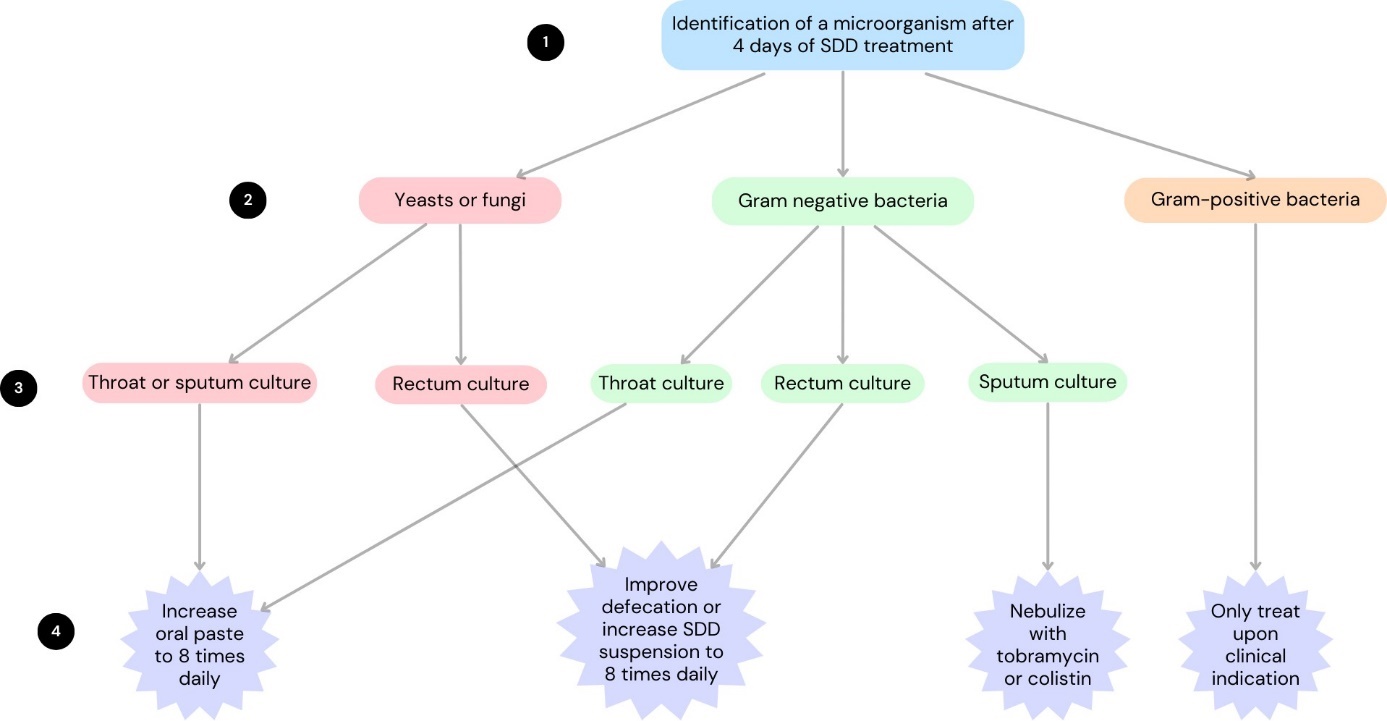


1: Detection of a microorganism after 4 days of SDD application; 2: type of microorganism identified; 3: culture in which the microorganism was found; 4: consequence for the SDD regimen

**Table S1. Missed potentially pathogenic microorganisms in scenarios B and C compared to scenario A (reference)**

|  | **Scenario B** | **Scenario C** |
| --- | --- | --- |
| ***Species*** | *Count per 1,000 ICU days* | *Count per 1,000 ICU days* |
| *Candida glabrata* | 1.17 | 3.03 |
| *Pseudomonas aeruginosa* | 1.05 | 2.43 |
| *Enterobacter cloacae complex* | 0.73 | 1.53 |
| *Escherichia coli* | 0.49 | 1.03 |
| *Stenotrophomonas maltophilia* | 0.28 | 1.61 |
| *Candida krusei* | 0.31 | 0.75 |
| *Acinetobacter baumannii/calcoaceticus group* | 0.18 | 0.45 |
| *Enterobacter aerogenes* | 0.14 | 0.32 |
| *Klebsiella pneumoniae* | 0.08 | 0.18 |
| *Achromobacter xylosoxidans* | 0.08 | 0.29 |
| *Morganella morganii* | 0.08 | 0.10 |
| *Proteus vulgaris complex* | 0.08 | 0.12 |
| *Serratia marcescens* | 0.06 | 0.18 |
| Other^1^ | 0.70 | 1.40 |

^1^ Species that were missed less frequently than 0.10 isolates per 1,000 ICU days in both scenarios are not reported in this table

**Table S2. Costs per SDD intensification trigger in scenario A and B for different subgroups^*^**

| **Subgroups^1^** | **ICU admissions**  **n (%)^2^** | **SDD intensification triggers^3^**  **n (%)^2^** | **Scenario A** | **Scenario B** |
| --- | --- | --- | --- | --- |
|  |  |  | **Costs per SDD intensification trigger^4^** | **Costs per SDD intensification trigger^4^** |
| **Complete cohort** | 7,231 (100) | 3,305 (100) | €1,523 (€1,473 – 1,579) | €1,503 (€1445 – 1566) |
| **Medical admissions** | 4,054 (56) | 1,957 (59) | €1,494 (€1,432 – 1,570) | €1,450 (€1,378 – 1,525) |
| **Acute surgical admissions** | 2,074 (29) | 855 (26) | €1,579 (€1,472 – 1,688) | €1,608 (€1,480 – 1,743) |
| **Elective surgical admissions** | 1,103 (15) | 493 (15) | €1,543 (€1,413 – 1,700) | €1,536 (€1,389 – 1,718) |
| **Immunocompromised patients** | 1,199 (17) | 611 (18) | €1,403 (€1,297 – 1,528) | €1,401 (€1,272 -1,554) |
| **Patients with COVID-19** | 463 (6) | 222 (6) | €1,151 (€1,044-€1,280) | €993 (€886-€1,117) |
| **Gastrointestinal surgery** | 344 (5) | 179 (5) | €1,386 (€1,212 – 1,612) | €1,310 (€1,118 – 1,557) |
| **Admission cultures positive** | 6,229 (86) | 3,082 (93) | €1,478 (€1,225 – 1,533) | €1,462 (€1,399 – 1,523) |
| **Admission cultures negative** | 1,002 (14) | 223 (7) | €2,143 (€1,893 – 2,470) | €2,100 (€1,817 – 2,479) |
| **First surveillance culture set positive^5^** | 2,191 (30) | 2,741 (83) | €1,018 (€980 – 1,058) | €862 (€826 – 900) |
| **First surveillance culture set negative^5^** | 773 (11) | 393 (12) | €1,877 (€1,700 – 2,081) | €1,737 (€1,558 – 1,985) |

^*^ Costs per detected SDD intensification trigger is undefined in Scenario C as no triggers were identified in SDD admission samples

^1^ With available culture costs data.

^2^ Relative to the complete cohort with available culture costs data.

^3^ SDD intensification triggers detected in SDD cultures only

^4^ Average costs for all SDD cultures (+ 95% CI), indexed to 2023 tariffs divided by the number of SDD intensification triggers detected in these SDD cultures.
^5^ In the subgroup of patients with at least two surveillance culture sets (culture set = throat, sputum, rectum).

**Supplementary analysis: COVID-19 period**

To test the robustness of our findings in a different clinical context and time period, we conducted a sensitivity analysis focused on ICU admissions during the COVID-19 pandemic (March 2020 to December 2022, n = 2,000). The results were highly comparable to those of the main analysis, suggesting that the observed effects are consistent across varying time periods and clinical settings.

*Primary findings*

Scenario A yielded 891 (95% CI 879-902) PPMs per 1,000 ICU days. In scenarios B and C, detection rates were 725 (95% CI 715-735) and 559 (95% CI 551-568) PPMs per 1,000 ICU days, respectively. In scenario A, 88 (95% CI 82-93) PPMs per 1,000 ICU days (9.9%) were deemed clinically relevant, including 6 (95% CI 5-7) multidrug-resistant PPMs (direct relevance), 66 (95% CI 62-70) PPMs resistant to standard therapy (high relevance), and 16 (95% CI 14-18) infection-related microorganisms. In scenarios B and C, 83 (95% CI 77-88) and 75 (95% CI 69-80) clinically relevant PPMs per 1,000 ICU days were detected, corresponding to 94% and 85% of clinically relevant PPMs identified in scenario A.

*Delayed detections*

Most of the clinically relevant PPMs identified in scenario A were detected on the same day in scenarios B and C. However, in scenario B, 2.4 (95% CI 1.9-3.2) clinically relevant PPMs per 1,000 ICU days were detected with a median delay of 3.5 (IQR 3-4) days, compared to 2.5 (95% CI 1.7-3.2) PPMs with a median delay of 3 (IQR 2-6) days in scenario C.

*Secondary outcomes*

In scenario A, there were 59 (95% CI 56–63) cases of colonisation persistence per 1,000 ICU days that required SDD intensification. Most (87%) were detected through SDD surveillance samples. In scenarios B and C, we observed 47 (95% CI 44–50) and 19 (95% CI 17–21) such triggers per 1,000 ICU days, respectively. Of the triggers identified in scenario A, 56.8% were detected on the same day in scenario B, while an additional 22.3% were identified with a median delay of 3 (IQR 2–5) days. In scenario C we observed 12.7% of triggers on the same day as in scenario A and an additional 19.6% with a median delay of 4 (IQR 3–5) days.

Cost data were available for 1,994 ICU admissions, representing 99.7% of the total cohort. The median expenditure for microbiological cultures was €833 (IQR €451-1583) per ICU admission, (€498 (IQR €304-871) for SDD cultures and €277 (IQR €90-802) for clinical cultures). The median costs per SDD culture were higher for admission samples than for surveillance samples (€65 (IQR €54-133) versus €54 (IQR €40-68), respectively). The total expenditure for SDD surveillance was €71,416 (95% CI €70,921-71,909) per 1,000 days in scenario A, compared to €49,073 (95% CI €48,636-49,532) and €27,191 (95% CI €26,824-27,571) in scenarios B and C, respectively; representing overall cost reductions of 31% and 62%. The cost per clinically relevant isolate — analogous to a “number needed to treat”— was €1,444 (95% CI €1,364-1,541) in scenario A, €1,216 (95% CI €1,137-1,305) in scenario B, and €822 (95% CI €768-887) in scenario C.
